# The immunomodulatory anticancer agent, RRx-001, induces an interferon response through epigenetic induction of viral mimicry

**DOI:** 10.1186/s13148-017-0312-z

**Published:** 2017-01-19

**Authors:** Hongjuan Zhao, Shoucheng Ning, Rosalie Nolley, Jan Scicinski, Bryan Oronsky, Susan J. Knox, Donna M. Peehl

**Affiliations:** 1 Department of Urology, Stanford University School of Medicine, 300 Pasteur Drive, Stanford, CA 94305 USA; 2 Department of Radiation Oncology, School of Medicine, Stanford University, 300 Pasteur Drive, Stanford, CA 94305 USA; 3 EpicentRx, Inc., 800W El Camino Real, Suite 180, Mountain View, CA 94040 USA

**Keywords:** Epigenetics, Cancer, RRx-001, Interferon response, Viral mimicry, Pseudo-infection

## Abstract

**Background:**

RRx-001, a dinitroazetidine derivative, is a novel anticancer agent currently in phase II clinical trials. It mediates immunomodulatory effects either directly through polarization of tumor associated macrophages or indirectly through vascular normalization and increased T-lymphocyte infiltration. With multiple additional mechanisms of action including upregulation of oxidative stress, depletion of GSH and NADPH, anti-angiogenesis and epigenetic modulation, RRx-001 is being studied as a radio- and chemo-sensitizer to resensitize tumors to prior therapy and to prime tumors to respond to radiation, chemotherapy and immunotherapy in combination therapy studies. Here, we identified another mechanism, viral mimicry, which refers to the “unsilencing” of epigenetically repressed viral genes present in the tumor that provokes an immune response and may contribute to the anticancer activity of RRx-001.

**Results:**

RRx-001 inhibited the growth of colon cancer cells (HCT 116) and decreased levels of the DNA methyltransferases DNMT1 and DNMT3a in a time and dose-dependent manner. Treatment of HCT 116 cells with 0.5 μM RRx-001 for 24 h significantly increased transcripts of interferon (IFN)-responsive genes and this induction was sustained for up to 4 weeks after transient exposure to RRx-001. ELISA assays showed that RRx-001 increased secretion of type I and III IFNs by HCT 116 cells, and these IFNs were confirmed to be bioactive. Transcription of endogenous retrovirus ERV-Fc2 and LTRs from the ERV-L family (MLT2B4 and MLT1C49) was induced by RRx-001. The induction of ERV-Fc2-env was through demethylation of ERV-Fc2 LTR as determined by methylation-specific polymerase chain reaction and combined bisulfite restriction analysis. Immunofluorescence staining with J2 antibody confirmed induction of double-stranded RNA.

**Conclusions:**

Transient exposure of HCT 116 cells to low-dose RRx-001 induced transcription of silenced retroviral genes present in the cancer cell DNA with subsequent synthesis of IFN in response to this “pseudo-pathogenic” stimulus, mimicking an antiviral defense. RRx-001-mediated IFN induction may have the potential to improve the efficacy of immunotherapies as well as radiotherapy, standard chemotherapies and molecularly targeted agents when used in combination. The striking safety profile of RRx-001 in comparison to other more toxic epigenetic and immunomodulatory agents such as azacitidine makes it a leading candidate for such clinical applications.

## Background

Immunotherapy has shown remarkable anticancer activity, with several immunotherapeutic modalities already approved by the Food and Drug Administration (FDA) for treating cancer patients [1]. Nevertheless, current immunotherapies only benefit 20–30% of patients in susceptible tumor types such as melanoma, non-small cell lung cancer and renal cell carcinoma. An emerging theme is that a relatively small percentage of treated patients demonstrate complete pathological remission or durable response, stimulating great interest in combining immunotherapy with chemotherapy and radiation [2, 3]. An even more attractive strategy to further improve the efficacy of cancer immunotherapy and to increase long-lasting disease control is to combine immunotherapy with agents that improve innate and/or adaptive immune responses. Along this line, of the many possible strategies being explored, combining immunotherapy with epigenetic agents is believed to hold considerable promise because of the well-established immunomodulatory potential of epigenetic drugs [4]. As discussed in a recent news feature in *Nature Medicine*, multiple global trials are underway to test combinations of immune checkpoint inhibitors and epigenetic therapies in solid tumors [5]. The epigenetic drugs include FDA-approved inhibitors of histone deacetylases (e.g., entinostat) and DNA methylation [e.g., azacitidine (5-AZA) and decitabine (5-AZA-CdR)] [4, 5].

A new paradigm to restore impaired immune function in tumors is the induction of viral mimicry by DNA methyltransferase inhibitors [6]. Viral mimicry is a term that, in an epigenetic context, refers to the transcription of silenced “endogenized” viral genes present in the tumor DNA and subsequent translation of viral proteins in the absence of an actual infection, which serves to excite a “vaccine-like” host immune response. Roulois et al. demonstrated that transient treatment of colon cancer cells with 5-AZA-CdR increased the levels of cytoplasmic double-stranded RNA (dsRNA) from endogenous transcripts including human endogenous retroviruses (ERVs), which in turn induced the expression of interferon (IFN)-stimulated genes (ISGs), including type III IFNs [7]. One striking consequence of this induction of viral mimicry by 5-AZA-CdR was the decreased frequency of cancer-initiating cells [7]. Similarly, Chiappinelli et al. showed that 5-AZA and 5-AZA-CdR triggered cytosolic sensing of dsRNA in ovarian cancer cells, causing a type I IFN response and apoptosis [8]. These studies pointed to a major role of the innate immune response in the antitumor effect of DNA demethylating agents. Moreover, because IFNs have been implicated in promoting strong immune responses, it is not surprising that in these studies, 5-AZA and 5-AZA-CdR sensitized checkpoint inhibitor-based immunotherapy in preclinical cancer models [7, 8].

In this study, we compared the novel immunomodulatory anticancer agent RRx-001 to 5-AZA in terms of ability to induce viral mimicry. Previously, we have shown that RRx-001 generates reactive oxygen and nitrogen species and nitric oxide, elicits changes in intracellular redox status, modulates tumor blood flow, hypoxia and vascular function, and triggers apoptosis in cancer cells [9–12]. For example, when administered to mice as a single agent, RRx-001 exhibited greater cytotoxicity than cisplatin or tirapazamine under hypoxic conditions [9]. When compared with cisplatin, RRx-001 was better tolerated at submaximal doses, yielding significant tumor growth inhibition in the absence of systemic toxicity [9]. Moreover, RRx-001 significantly decreased DNMT1 and DNMT3A protein expression in a dose- and time-dependent manner in murine SCC VII cancer cells and induced demethylation of genes important in pathways relevant to cancer [13]. Finally, RRx-001 activates Nrf2-ARE antioxidant signaling pathways in tumor cells and therefore measurement of Nrf2-mediated activation of downstream target genes through ARE signaling may constitute a useful molecular biomarker for the early prediction of response to RRx-001 treatment and thereby guide therapeutic decision-making [10].

Currently, RRx-001 is undergoing evaluation as a single chemotherapeutic agent to resensitize tumors to prior therapy and to prime tumors to respond to radiation, chemotherapy and immunotherapy in combination therapy studies [9, 12, 13] and in phase II clinical trials (https://clinicaltrials.gov/ct2/show/NCT02096354?term=epicenTRX&rank=1, https://clinicaltrials.gov/ct2/show/NCT02489903?term=epicenTRX&rank=4, https://clinicaltrials.gov/ct2/show/NCT02452970?term=epicenTRX&rank=3). These clinical trials have demonstrated the clear-cut safety advantage of RRx-001, which, unlike 5-AZA and other epigenetic therapies, is non-myelosuppressive and has minimal to no systemic toxicity [14, 15]. Likewise, despite the therapeutic potential of checkpoint inhibitors such as ipilimumab or nivolumab, immune-related adverse events such as diarrhea are not uncommon with these agents, occurring in 16–27% of treated patients and in 55% when used in combination [16]; in contrast, the benign toxicity profile of RRx-001 [14] facilitates combination with other therapies. We examined the effects of transient exposure of HCT 116 cells to RRx-001 versus 5-AZA on expression of IFNs and ISGs as well as dsRNAs and ERVs, and whether these effects were mediated by DNA demethylation. We found that transient exposure of HCT 116 cells to low-dose RRx-001 induced transcription of silenced retroviral genes present in the cancer cell DNA with subsequent synthesis of IFN and ISGs in response to this “pseudo-pathogenic” stimulus, mimicking an antiviral defense.

## Methods

### Reagents

RRx-001 was obtained from Orbital ATK (Dulles, VA, USA). The DNA methyltransferase inhibitor 5-azacytidine (5-AZA) was purchased from Sigma-Aldrich (St. Louis, MO, USA). Ruxolitinib was purchased from Selleck Chemical (Houston, TX, USA). RRx-001, 5-AZA and ruxolitinib solutions were freshly prepared on the experimental day by dissolving in DMSO and then diluting with cell culture medium. The final concentration of DMSO was 0.025%.

### Cell culture and treatments

Human HCT 116 cells (gift of Dr. Calvin Kuo at Department of Medicine, Stanford University, Stanford, CA, USA) were authenticated by the Genomic Resources Core Facility (GRCF) at John Hopkins University (Baltimore, MD, USA) using a short-tandem repeat (STR) analysis. The cells were also determined to be mycoplasma negative by GRCF. Murine SCC VII and human HCT 116 cells were grown in DMEM medium supplemented with 10% fetal calf serum and 100 μg/ml gentamycin in a 37 °C humidified incubator with a mixture of 95% air and 5% CO_2_. Exponentially growing cells were treated with 5-AZA, RRx-001, or 0.025% DMSO as a vehicle control for 1 to 3 days. Media containing RRx-001, 5-AZA, or DMSO were replaced daily over the 3-day period.

### In vitro cytotoxicity assay

Cell counting kit-8 (CCK-8), a modified MTT assay with 2-(2-methoxy-4-nitrophenyl)-3-(4-nitrophenyl)-5-(2,4-disulfophenyl)-2H-tetrazolium (Dojindo Molecular Technologies, Rockville, MD, USA), was used to measure the cytotoxicity of RRx-001. Cells were inoculated into 96-well plates and incubated overnight. Freshly prepared solutions of RRx-001 were added in the final concentrations ranging from 0.1 to 1000 μM in 200 μL of loading volume. Three days later, plates were washed with phosphate-buffered saline (PBS) and refilled with 100 μL of phenol red-free growth medium. CCK-8 solution was added and after a 2-h incubation at 37 °C, the optical absorbance of wells was measured at a wavelength of 450 nm with a microplate reader (Molecular Devices, Sunnyvale, CA, USA). Cell viability was calculated as the percentage of the optical density of the treated cells to that of untreated control cells.

### Western blots

After treatment with DMSO, 0.5 μM 5-AZA, or 0.5-5 μM RRx-001 for 1–3 days, cells were washed twice with cold PBS and lysed in RIPA buffer (Sigma-Aldrich, St. Louis, MO, USA) for preparation of whole cell lysates. Medium was changed daily with fresh drug during treatment. Protein contents were quantified using a Bio-Rad Quick Start protein assay kit (Bio-Rad Laboratories, Hercules, CA, USA). Samples containing equal amounts of total protein (20 μg) were separated on 10% SDS-PAGE gels and transferred onto PVDF membranes. The membranes were blocked with 5% non-fat milk and probed with primary antibodies against DNMT1 (1:500, Cell Signaling Technology, Danvers, MA, USA), DNMT3a (1:500, Santa Cruz Biotechnology, Santa Cruz, CA, USA) and β-actin (1:500, Santa Cruz Biotechnology), and HRP-conjugated secondary antibodies (1:2500, Santa Cruz Biotechnology). The immunoreactive proteins were detected with ECL plus chemiluminescence detection reagents (Amersham Biosciences, Little Chalfont, UK). The Western blot analyses were run at least twice for each molecular marker, unless otherwise indicated.

### Quantitative polymerase chain reaction (qPCR)

RNA was isolated using Trizol reagent according to the manufacturer’s instructions (Invitrogen, Carlsbad, CA, USA. Two micrograms of DNase-treated RNA were used to synthesize cDNA using PrimeScript RT reagent kit (Takara, Mountain View, CA, USA), and one twentieth of the resulting cDNA was subjected to PCR amplification using SYBR Green PCR Master Mix (Invitrogen). The level of RPLP0 RNA was used as an internal control to normalize expression levels of genes of interest. PCR amplification was performed on the Mx3005P QPCR system (Stratagene, San Diego, CA, USA). Primers used in this study are listed in Table 1.

**Table 1:** Primers used in qPCR

Gene name	5′->3′ primer	3′->5′ primer
DDX58	CCAGCATTACTAGTCAGAAGGAA	CACAGTGCAATCTTGTCATCC
Env-Fc2	CTCCATTAGTAGCAGTTCCTCTCC	GAGAATAGTGGGACCTGTCCTTT
IFI27	ACCTCATCAGCAGTGACCAGT	ACATCATCTTGGCTGCTATGG
IFI44	GAGAGATGTGAGCCTGTGAGG	TTTTCCTTGTGCACAGTTGAT
IFI44L	GTGGATGATTGCAGTGAGGTT	AATATCCTTCATGGGGTCCAG
IFI6	TGCTACCTGCTGCTCTTCAC	CGAGCTCTCCGAGCACTTTT
IL28A	TCCAGTCACGGTCAGCA	CAGCCTCAGAGTGTTTCTTCT
IL29	GAAGACAGGAGAGCTGCAACGTGGA	GGTTCAAATCTCTGTCACCACA
IRF7	CTGAGGGCTTGTAG	TCAACACCTGTGACTTCATGT
ISG15	GCCTCAGCTCTGACACC	CGAACTCATCTTTGCCAGTACA
MLT1C49	TATTGCCGTACTGTGGGCTG	TGGAACAGAGCCCTTCCTTG
MLT2B4	GGAGAAGCTGATGGTGCAGA	ACCAACCTTCCCAAGCAAGA
OASL	GCAGAAATTTCCAGGACCAC	CCCATCACGGTCACCATTG
RPLP0	CAGACAGACACTGGCAACA	ACATCTCCCCCTTCTCCTT
Env-Fc2 methylated	CGGGCGAGGTTTATTGGTTCG	CGCYTATTCCCWTTTATTCCCG
Env-Fc2 unmethylated	TGGGTGAGGTTTATTGGTTTGTAGG	CACYTATTCCCWCCATTTATTCCCA
Env-Fc2 COBRA	GTATAATGGGGAAAAATGGAA	ACTTCCCTAACCCTCCC

### ELISA

Levels of type I IFN (IFN-β) and type III IFN (IL-29/IL-28B) were determined using a VeriKine Human IFN-beta ELISA Kit (PBL Assay Science, Piscataway Township, NJ, USA) and a Human IL-29/IL-28B (IFN-lambda 1/3) DuoSet ELISA Kit (R&D Systems, Minneapolis, MN, USA) according to manufactures’ instructions, respectively. Fifty microliters of media were used for each well and triplicates were evaluated for each sample.

### Immunofluorescence staining

Cells were fixed with 2% paraformaldehyde in PBS for 10 min, permeabilized with 0.5% Triton X-100 in PBS for 10 min, and blocked with 10% horse serum in PBS for 1 h. Cells were then stained with primary antibody against double-stranded RNA (J2, 1:200, Scicons, Szirák, Hungary), and goat anti-mouse Alexa 555-conjugated secondary antibody (1:1000, Invitrogen). To quantify J2-negative cells, five random fields were selected and J2-negative cells were counted.

### Methylation assays

Genomic DNA was bisulfite-converted using the EZ DNA Methylation Kit (Zymo Research, Irvine, CA, USA). Methylation-specific PCR was performed as previously described for the ERV-Fc2-env gene [8]. COBRA (Combined Bisulfite Restriction Analysis) was performed at the ERV-Fc2 LTR located on chromosome 11 as previously described [8]. Bisulfite-treated DNA was amplified and digested with the AciI enzyme. Unmethylated DNA was not digested and remained intact as a 199 bp band. Methylated DNA was digested into two bands (155 and 44 bp). Bands and restriction fragments were resolved on 2% agarose gels, stained with ethidium bromide, and photoimaged with a gel imaging system. Signal intensity was quantified using ImageJ software (https://imagej.net). Primers for methylation-specific PCR and COBRA are listed in Table 1.

#### Statistical analysis

Two-tailed Student’s *t* test was used for two-arm experiments. A *p* < 0.05 was considered significant. Statistical tests were performed with Excel Stats.

## Results

### RRx-001 inhibited growth of HCT 116 cells

**Fig. 1:**
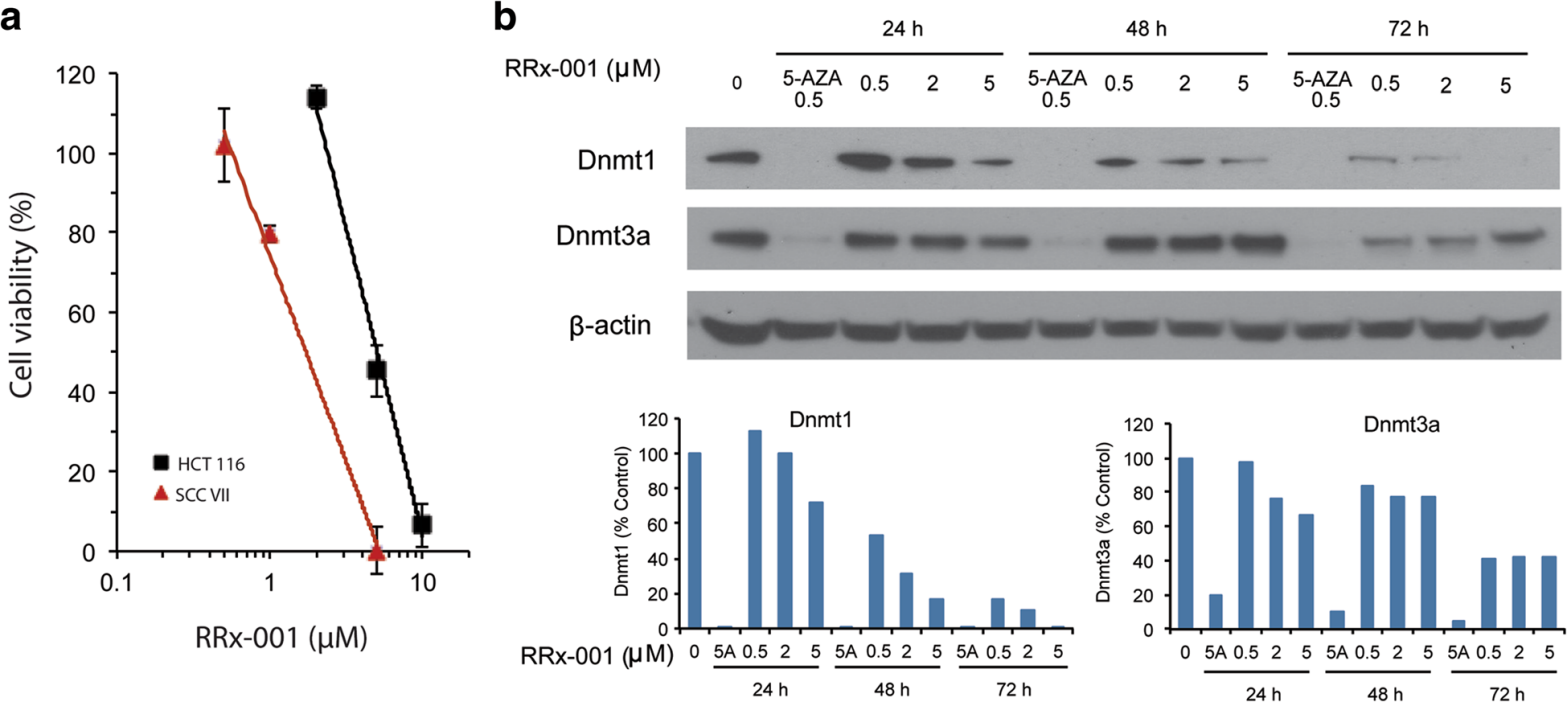
RRx-001 inhibited cell growth and DNMT1 and DNMT3a expression in HCT 116 cells. Exponentially growing cells were treated with 5-AZA, RRx-001 or 0.025% DMSO as a vehicle control for 1 to 3 days. Media containing RRx-001, 5-AZA, or DMSO were replaced daily over the 3-day period. **a** Concentration-survival curve of HCT 116 cells treated with RRx-001. SCC VII cells were used as a positive control. Data are mean ± SD from 3 independent experiments. **b** Protein levels of DNMT1 and DNMT3a in HCT 116 cells treated with RRx-001 as determined by Western blot. β-actin was used as an internal control for sample loading. 5-AZA was used as a positive control for inhibition of DNMT1 and DNMT3a expression. *Bar graphs* show the relative expression of DNMT1 and DNMT3a in treated cells compared to control cells

In vitro antiproliferative activity of RRx-001 was previously demonstrated in a panel of 12 cancer cell lines, with IC_50_ values ranging from 1.8–6.0 μM [9]. Using the same cytotoxicity assay as in the previous study [9], we found that HCT 116 colon cancer cells showed an IC_50_ of 5.03 μM in response to RRx-001, whereas SCC VII (murine squamous cell carcinoma), the most responsive cell line among the 12 cancer cell lines previously tested, had an IC_50_ of 1.8 μM (Fig. 1a). These results demonstrated that HCT 116 cells were responsive to the growth-inhibitory activity of RRx-001.

### RRx-001 downregulated DNMT1 and DNMT3a proteins in HCT 116 cells

We previously showed that RRx-001 significantly decreased protein levels of the DNA methyltransferases DNMT1 and DNMT3a in SCC VII cells in a dose- and time-dependent manner [13]. Here, we investigated the effect of RRx-001 on DNMT1 and DNMT3a in HCT 116 cells. Figure 1b shows protein levels of the two DNA methyltransferases in cells treated with 0.5 μM 5-AZA or 0.5–5 μM RRx-001 for 24–72 h. As anticipated, the levels of both proteins were dramatically decreased by 0.5 μM 5-AZA as early as 24 h after treatment, as reported in previous publications [17]. RRx-001 also decreased levels of DNMT1 and DNMT3a in a time and dose-dependent manner, confirming that RRx-001 is a DNA methyltransferase inhibitor. However, downregulation of DNMTs by RRx-001 required higher concentrations and longer exposure compared to 5-AZA. Whereas DNMT1 decreased to <5% of control by 24 h after treatment with 0.5 μM 5-AZA, equivalent reduction of DNMT1 by RRx-001 did not occur until 72 h after treatment with 5 μM RRx-001. DNMT3a was also reduced to a lesser extent by RRx-001 compared to 5-AZA. Altogether, these results suggest that there are both similarities and differences in the mechanisms of action of these two drugs.

### Transient exposure of HCT 116 cells to RRx-001 resulted in sustained induction of ISGs

**Fig. 2:**
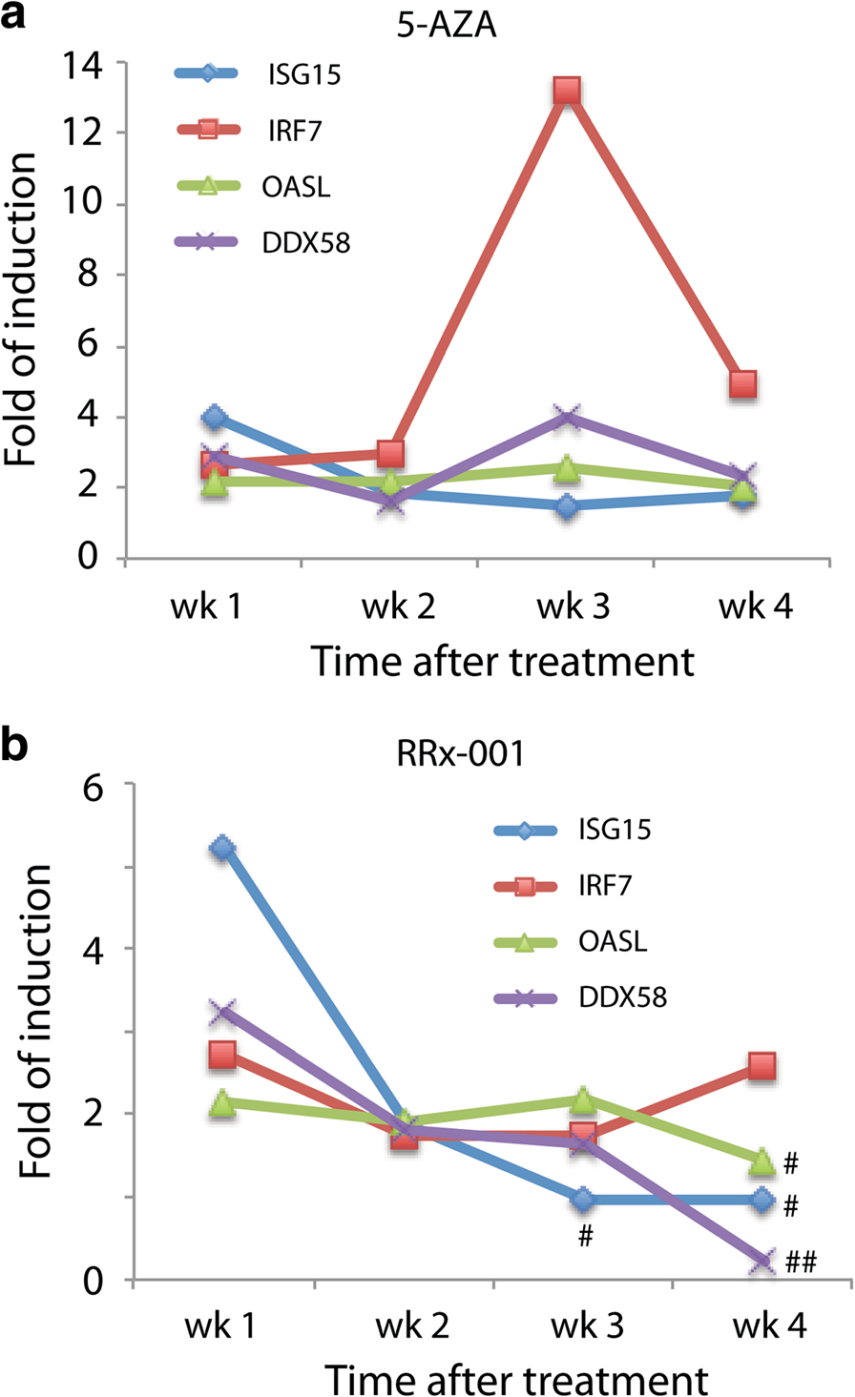
Transient treatment with RRx-001 induced a sustained IFN response in HCT 116 cells. Expression of IFN-stimulated genes (ISGs) over the course of 4 weeks in response to 24 h of treatment with 0.5 μM 5-AZA (**a**) or RRx-001 (**b**). Transcript levels of ISGs (*ISG15*, *IRF7*, *OASL*, and *DDX58*) were determined by qPCR 7 days after treatment. Upregulation of gene expression observed in 5-AZA- treated cells was statistically significant at all time points. Gene expression changes observed in RRx-001-treated cells that were not significantly different from DMSO-treated cells were marked by # and those that were significantly downregulated by ##

The ability of RRx-001 to induce four selected ISGs (*IRF7, OASL*, *ISG15*, and *DDX58*) in HCT 116 cells was compared to that of 5-AZA. Cells were transiently (24 h) treated with 0.5–5 μM RRx-001 or 0.5 μM 5-AZA and subsequently maintained in drug-free medium for an additional 7 days. RNA transcripts of all 4 genes were significantly increased by 0.5 μM 5-AZA as measured by qPCR (Fig. 2a), reproducing previous findings [7, 8]. Notably, 0.5 μM RRx-001 also significantly increased transcripts of all 4 genes, to levels equivalent to or even higher than induced by 5-AZA (Fig. 2b). Induction of the 4 genes by 2 μM RRx-001 tended to be less than that with 0.5 μM, and none of the transcripts were significantly increased by 5 μM RRx-001 (data not shown), similar to our previous finding that RRx-001 induced epigenetic effects at lower rather than higher concentrations [13]. In this regard, RRx-001 is similar to 5-AZA, which in some cases has elicited a greater effect on methylation at lower concentrations [18, 19].

To determine whether the effect of RRx-001 on expression of ISGs in HCT 116 cells was sustained, we cultured the cells for 4 weeks after transient (24 h) exposure to 0.5 μM RRx-001 and quantified transcripts of the 4 ISGs at weeks 2, 3, and 4. Cells transiently treated with 0.5 μM 5-AZA were similarly maintained and evaluated. As shown in Fig. 2a, all 4 ISGs remained at significantly elevated levels at weeks 2, 3, and 4 after transient exposure to 5-AZA. All 4 genes at week 2, and 3 of 4 genes at week 3, remained elevated after transient exposure to RRx-001 (Fig. 2b), whereas only 1 of 4 genes still showed significantly increased expression at week 4 (Fig. 2b). These results showed that transient treatment with RRx-001 resulted in sustained elevated expression of ISGs in HCT 116 cells, albeit with a less long-lasting effect than that of 5-AZA.

### RRx-001 induced bioactive type I and type III IFNs

**Fig. 3:**
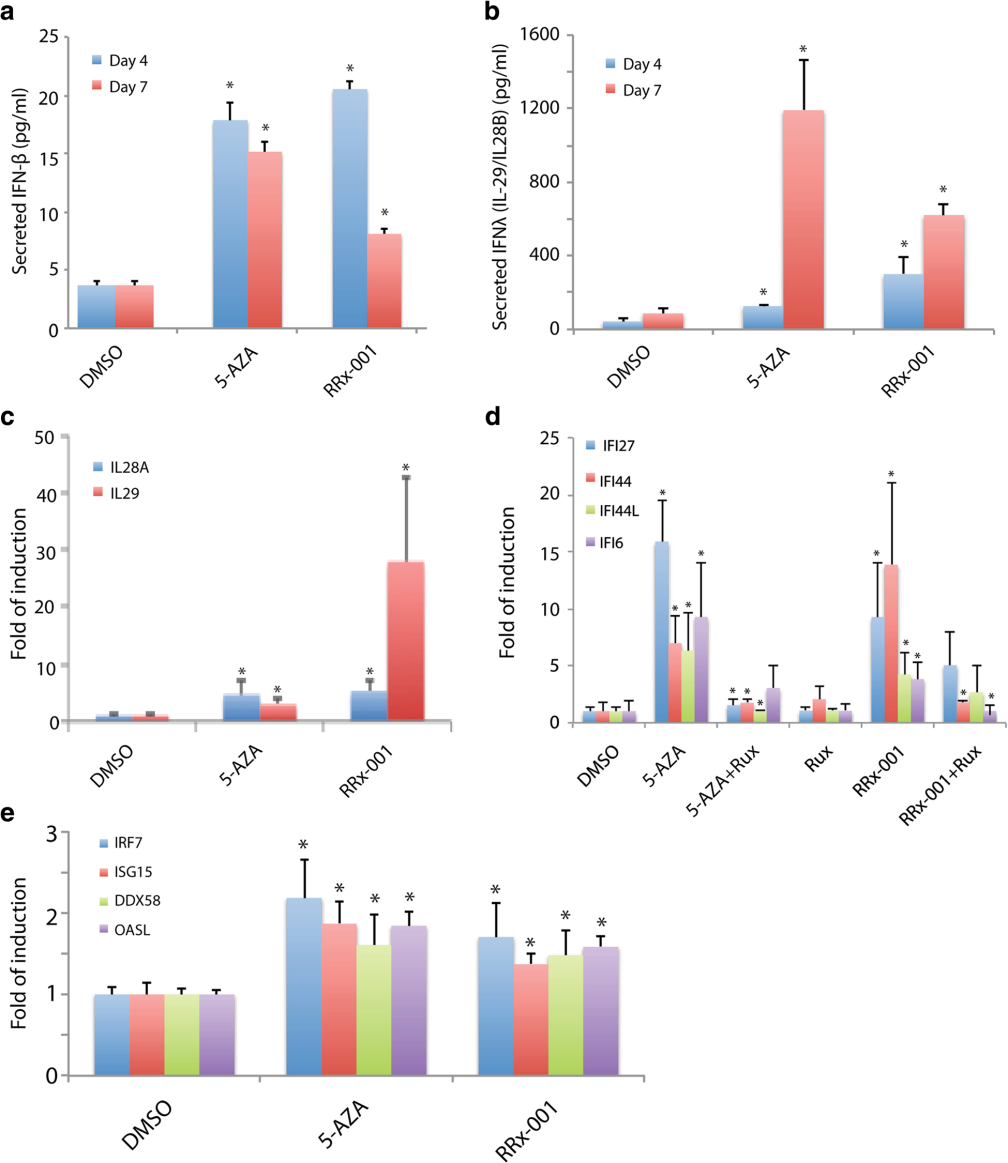
RRx-001 induced IFN response through upregulation of type I and III IFN expression and JAK/STAT pathway. Cells were transiently (24 h) treated with 0.5 μM RRx-001 or 0.5 μM 5-AZA and subsequently maintained in drug-free medium for an additional 7 days. RRx-001 induced a significant increase in type I IFN (IFN-β) (**a**) and type III IFN (IL-29/IL-28B) (**b**) secretion into culture medium by HCT 116 cells as measured by ELISA. Transcript levels of *IL29*/*IL28A* were also increased as determined by qPCR (**c**). ISGs (*IFI27*, *IFi44*, *IFI44L*, and *IFI6*) were upregulated by 5-AZA and RRx-001 but blocked by the JAK/STAT inhibitor ruxolitinib (rux) at 2 μM concentration (**d**). Expression of ISGs (*IRF7*, *ISG15*, *DDX58*, and *OASL*) was also upregulated in HCT 116 cells cultured in conditioned medium containing secreted IFNs induced by 5-AZA and RRx-001 as determined by qPCR (**e**)

To determine which IFNs might be responsible for the induction of ISGs by RRx-001 in HCT 116 cells, we examined RNA and protein levels of several type I and type III IFNs. Secreted protein levels of a type I IFN, IFNβ, were measured by ELISA after treatment of HCT 116 cells with 0.5 μM RRx-001 or 5-AZA. Both compounds significantly increased the amounts of IFNβ secreted 4 days after transient (24 h) treatment (Fig. 3a). Levels of secreted IFNβ remained significantly elevated 7 days after treatment with 5-AZA but not RRx-001 (Fig. 3a). Both compounds also significantly increased the amounts of type III IFN [IFNλ 1/3 (IL-29/IL-28B)] secreted 4 days after transient treatment (Fig. 3b). The induction of secreted IFNλ 1/3 was more prominent 7 days after treatment with 5-AZA or RRx-001 (Fig. 3b). Finally, transcripts of two type III IFNs, *IL28A* and *IL29*, showed a modest, but significant, increase after transient treatment with 5-AZA or RRx-001 (Fig. 3c). These results suggest that RRx-001 induced ISGs through upregulation of both type I and III IFNs, similarly to 5-AZA.

To determine whether induction of IFNs by RRx-001 involved JAK, cells were treated with ruxolitinib, a JAK1/JAK2 inhibitor, in conjunction with RRx-001 or 5-AZA. Ruxolitinib blocked the induction of ISGs by 5-AZA, as previously reported [7, 8], and also significantly blocked induction of 2 of 4 ISGs by RRx-001 (Fig. 3d). These results suggest that induction of IFNs and upregulation of ISGs in cells treated with RRx-001 is mediated by JAK.

To determine whether the secreted IFNs induced by 5-AZA and RRx-001 were bioactive, HCT 116 cells were cultured in medium previously conditioned by HCT 116 cells maintained for 7 days after transient exposure to 0.5 μM 5-AZA or RRx-001. Transcripts of the 4 ISGs were significantly increased in cells cultured in conditioned medium compared to control (Fig. 3e), indicating that the secreted IFNs induced by 5-AZA and RRx-001 were bioactive.

### RRx-001 induced dsRNA and LTRs from the ERV-L family in HCT 116 cells

**Fig. 4:**
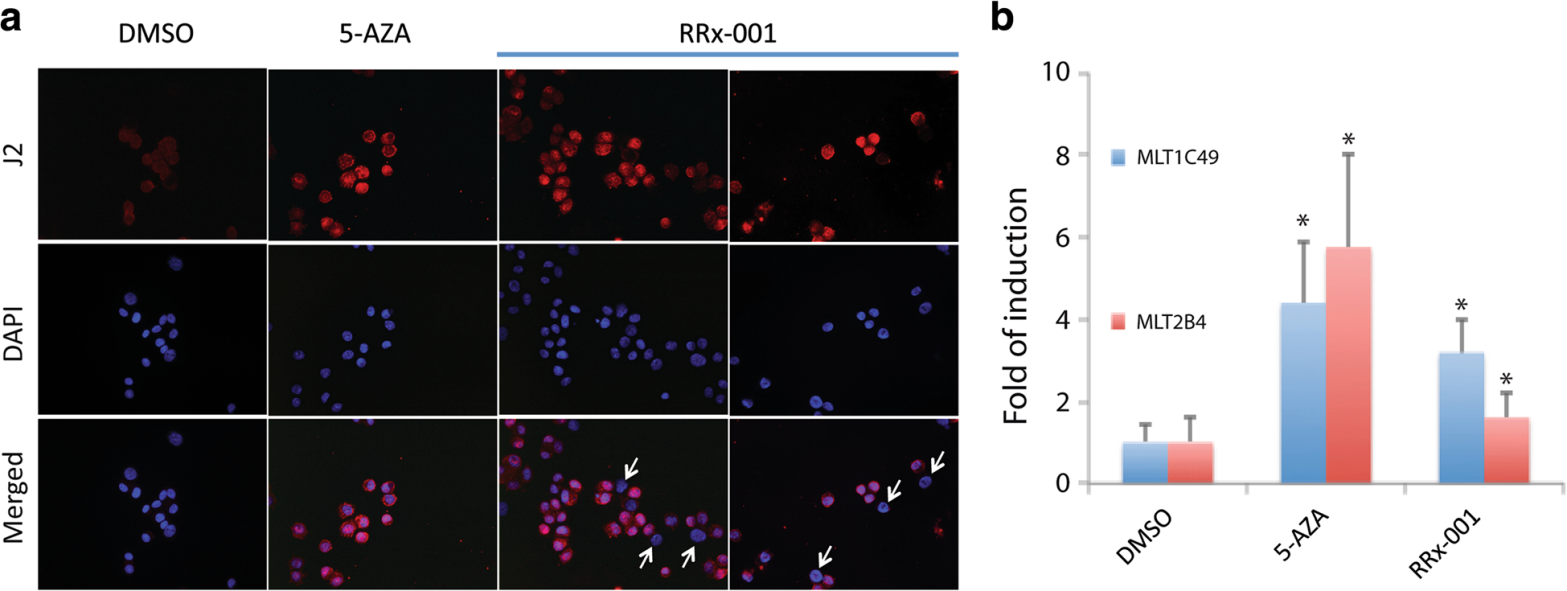
RRx-001 upregulated ERV transcription. Cells were transiently (24 h) treated with drugs and maintained for an additional 5 days in drug-free medium. **a** Immunofluorescence staining of dsRNA using J2 antibody (*red*) in DMSO-, 5-AZA-, or RRx-001-treated cells. DAPI was used to stain the nucleus (*blue*). *White arrows* point to cells that were negative for dsRNA. **b** Transcript levels of two ERVs were upregulated by 5-AZA and RRx-001 compared to DMSO as determined by qPCR

The J2 antibody was used to monitor the presence of dsRNA in HCT 116 cells after treatment with 5-AZA or RRx-001. Cells were transiently (24 h) treated with drugs and maintained for an additional 5 days in drug-free medium. 5-AZA uniformly increased immunofluorescence staining with J2 in ~100% of cells (Fig. 4a). RRx-001 similarly increased J2 staining in the majority of cells, indicating that RRx-001, like 5-AZA, induced expression of dsRNA (Fig. 4a). Notable, however, was the approximately 14% of J2-negative cells among the population treated with RRx-001 (arrows in Fig. 4a) but not with 5-AZA, suggesting that some HCT 116 cells were less responsive to the induction of dsRNA by RRx-001.

To determine whether the increase in dsRNA was attributable to increased expression of endogenous retroviruses (ERVs), we measured transcript levels of MTL1C49 and MTL2B4, two ERVs among those found to be most highly induced by 5-AZA treatment of HCT 116 cells in a previous study [7]. Indeed, expression of both ERVs was increased by 0.5 μM 5-AZA (Fig. 4b). An equivalent concentration of RRx-001 also significantly increased transcripts of both ERVs, although to somewhat lesser levels (Fig. 4b). These findings, together with the increase in J2 staining, show that RRx-001, like 5-AZA, induces expression of LTRs from the ERV-L family (MLT2B4 and MLT1C49) by transient treatment of HCT 116 cells.

### RRx-001 demethylated ERV-Fc2 LTR

**Fig. 5:**
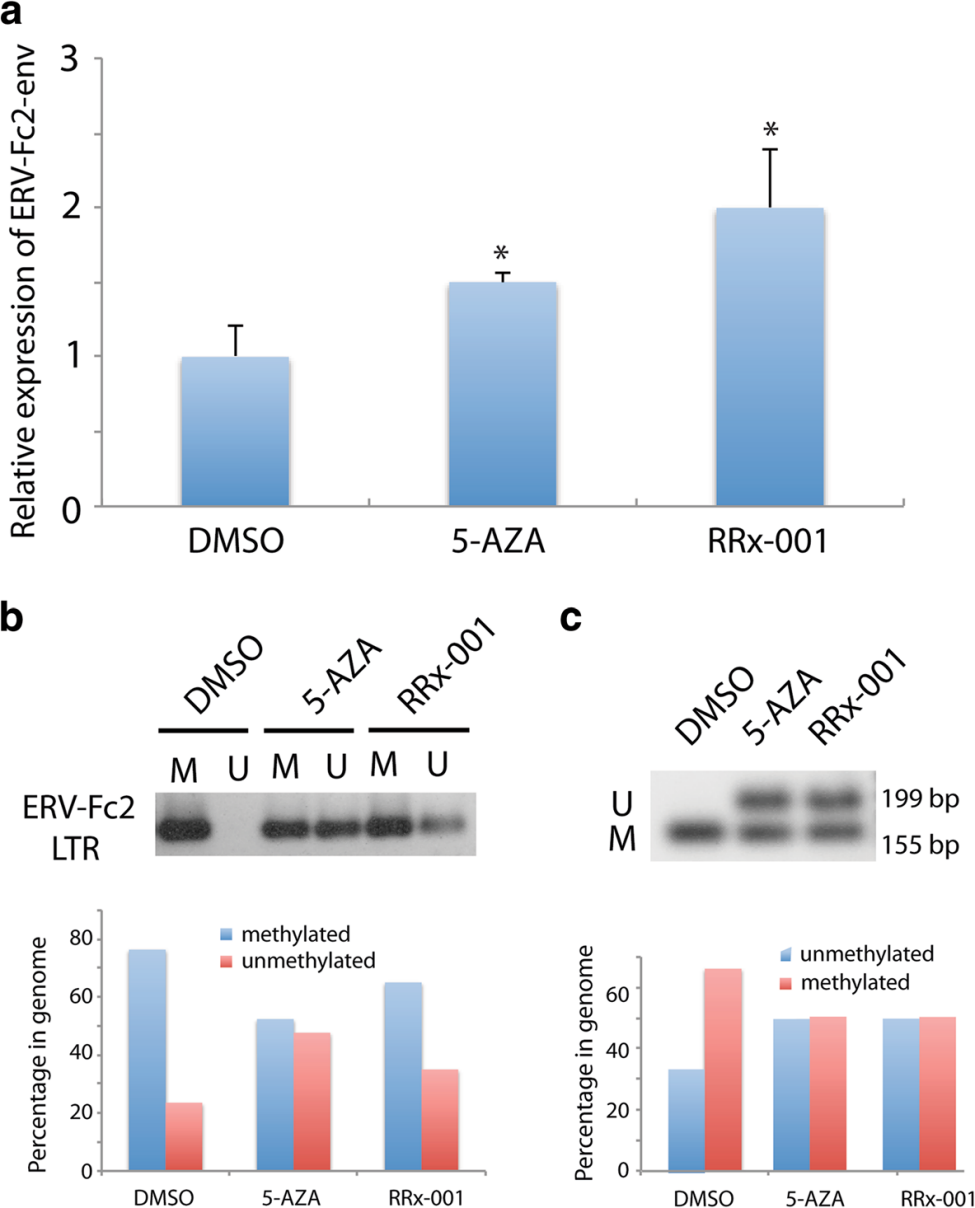
RRx-001 induced ERV-Fc2-env expression through DNA demethylation. Cells were transiently (24 h) treated with drugs and maintained for an additional 5 days in drug-free medium. **a** Transcript levels of ERV-Fc2-env were upregulated by 5-AZA and RRx-001 compared to DMSO as determined by qPCR. **b** Methylation of ERV-Fc2 LTR was decreased by 5-AZA and RRx-001 compared to DMSO as determined by methylation-specific PCR. **c** Methylation of ERV-Fc2 LTR was decreased by 5-AZA and RRx-001 compared to DMSO as determined by COBRA. Bisulfite-treated DNA was amplified and digested with the AciI enzyme. Unmethylated DNA (U) was not digested and remained intact as a 199 bp band. Methylated DNA (M) was digested into two bands (155 and 44 bp). Signal intensity was quantified using ImageJ software (https://imagej.net)

To determine whether induction of ERV expression by RRx-001 was mediated by DNA demethylation, we examined the methylation status of ERV-Fc2-env, a member of the extended ERV-F/ERV-H family. Cells were transiently (24 h) treated with drugs and maintained for an additional 5 days in drug-free medium. Consistent with the observed increase in ERV-Fc2-env transcripts after transient exposure to 0.5 μM RRx-001 or 5-AZA as determined by qPCR (Fig. 5a), treatment of HCT 116 with 5-AZA or RRx-001 resulted in increased levels of unmethylated DNA at the CpG loci of ERV-Fc2 LTR as measured by methylation-specific PCR (Fig. 5b) and COBRA (Fig. 5c). These results suggest that the induction of dsRNA by RRx-001 is mediated by DNA demethylation of ERV-Fc2 LTR.

## Discussion

One of the major problems with current anticancer modalities is a lack of specificity that often results in systemic and dose-limiting toxicities. The low inherent toxicity of RRx-001 and the selective red blood cell-mediated localization of its cytoactive metabolites in tumors [11] set RRx-001 apart from current anticancer modalities such as traditional chemotherapeutics, targeted small molecule therapies, radiation, epigenetic agents, and even checkpoint inhibitors. Several studies have shown that RRx-001 possesses diverse, pleiotropic mechanisms of action including reeducation of tumor associated macrophages from an M2 to an M1 phenotype, upregulation of oxidative stress, GSH and NADPH depletion, vascular normalization, and epigenetic modulation [9–13, 20] that may enhance the antitumor immune response. In this study in HCT 116 cells, we identified an additional mechanism underlying immunomodulatory activity of RRx-001, involving stimulation of a type I IFN response through transcriptional reactivation of remnant ERVs that are present in the tumor DNA. The translation of these viral proteins simulates an infection and an immune response to this “pseudo-infection” ensues. The induction of IFN signaling through the JAK-STAT pathway with RRx-001 was long-term and likely mediated by reduction of DNA methyltransferases DNMT1 and DNMT3a, which in turn led to demethylation of ERV-Fc2 LTR. Interestingly, in a dose-dependent inverse effect that is characteristic of epigenetic inhibitors, lower doses of RRx-001 were associated with greater induction of ISGs. This induction of a “pseudo-infected” state with the subsequent production of ISGs may serve to improve the efficacy of immunotherapies such as checkpoint inhibitor-based therapies recently approved by FDA including anti-CTL4 and anti-PD-1/PDL-1 as well as other small molecules and standard chemotherapies.

The ability of epigenetic drugs to improve treatment with immune checkpoint inhibitors has been demonstrated in a variety of preclinical models. Kim et al. showed that co-treatment with anti-CTLA-4 and epigenetic-modulating drugs, i.e., 5-AZA and entinostat, a class I HDAC inhibitor, markedly improved treatment outcomes, curing more than 80% of mice bearing colorectal or breast cancers [21]. In addition, combined treatment with anti-CTLA-4 and either 5-AZA-CdR [22] or the second-generation DNMT inhibitor guadecitabine [23] significantly reduced the growth of poorly immunogenic syngeneic grafts of murine mammary carcinoma TS/A and of mesothelioma AB1 with respect to treatment with the single agents. In the B16-F10 mouse melanoma model, multiple combinations of low-dose 5-AZA directly enhanced tumor responses to anti-CTLA4 immune checkpoint therapy [8]. These studies support the addition of epigenetic agents to immune checkpoint inhibitor therapies in clinical trials to increase their spectra of activity. Two examples of such clinical trials are the ongoing phase Ib trial NIBIT-M4 (EUDRACT 2015-001329-17), combining guadecitabine and an anti-CTLA-4 monoclonal antibody (ipilimumab) in metastatic melanoma, and the randomized phase II trial NCT01928576 combining an “epigenetic priming” with azacytidine/oral azacytidine, given alone or in association with entinostat, with anti-PD-1 immunotherapy in patients with non-small cell lung cancer. Interestingly, Liu et al. recently reported that vitamin C increases viral mimicry induced by low doses of 5-AZA-CdR in vitro, suggesting that correction of vitamin C deficiency in cancer patients may improve responses to epigenetic therapy with DNMT inhibitors [24].

Our study strengthens the rationale to combine RRx-001 with immunotherapy to enhance its efficacy. Similar to 5-AZA, RRx-001 is a DNA demethylating agent that inhibits DNMT1 and DNMT3a expression, but, unlike 5-AZA, it is non-myelosuppressive, which means that the dose modifications, interruptions, delays, reductions, and discontinuations due to hematologic adverse events, which are typical with 5-AZA, are unlikely with RRx-001 and, indeed, to date, have not been observed, resulting in comparatively longer-term treatment. The inhibition of DNMT1 and DNMT3a expression results in the demethylation of ERV-Fc2 LTR and the subsequent upregulation of IFN production by dsRNA sensing. In addition, Peng et al. showed that DNA methylation represses tumor production of CXCL9 and CXCL10 [25]. Treatment with epigenetic modulators removes the repression and increases effector T cell tumor infiltration, slows down tumor progression, and improves the therapeutic efficacy of PD-L1 checkpoint blockade and adoptive T cell transfusion in tumor-bearing mice [25]. Moreover, stimulation of IFN response has been shown to be one of the major mechanisms by which chemotherapeutic drugs enhance anticancer immune responses [26, 27]. The intrinsic antitumor effects of IFNs include regulating the expression of many genes that directly affect tumor cell proliferation, differentiation, survival, migration, and other specialized functions and upregulating tumor antigens on tumor cells as well as antigen presentation by major histocompatibility complexes (MHCs) [28, 29]. In addition, IFNs exert extrinsic antitumor effects by regulating the activity of almost all immune cell types (including macrophages, dendritic cells, B cells, T cells, and innate lymphocytes), creating a well-orchestrated immune response to counter infectious and malignant disease [29]. Given their pleiotropic actions, it is not surprising that the intratumoral expression levels of IFNs or of ISGs correlated with favorable disease outcome in several cohorts of patients with cancer [28]. However, IFN signaling is known to induce a compensatory upregulation of immunosuppressive cytokines and receptors [30], which perhaps lends a note of caution to the use of IFN-stimulating agents in cancer.

A number of important characteristics of RRx-001 make it an attractive partner for immunotherapy. First, in addition to its remarkably benign safety profile in human and animal studies [14], emerging data suggest that RRx-001 may also protect normal tissues against some of the deleterious side effects of immuno-, chemo- or radiotherapy, such as diarrhea and myelosuppression, thereby improving therapeutic index. Second, the lack of immune cell infiltration into tumors has been cited as one reason for failure of immunotherapy, and normalization of vasculature may improve trafficking of T cells into tumors [31]. RRx-001 has been shown to improve vascularity of murine tumors by redistributing blood flow [32]. Finally, HDAC inhibitors promote antitumor immunity and are being tested in clinical trials in combination with checkpoint inhibitors. Although not explored in this study, we have preliminary evidence that RRx-001 is an inhibitor of histone deacetylases as well as of DNA methyltransferases [13]. Taken together, this study suggests that RRx-001 via multiple mechanisms including epigenetic inhibition may improve immune activity, supporting its development as a primer to potentiate the efficacy of currently available anticancer modalities including checkpoint inhibitor-based immunotherapy.

## Conclusions

RRx-001 is an immunomodulatory anticancer agent with multiple mechanisms of action, including induction of interferon-stimulated genes through viral mimicry, which refers to the establishment of a “pseudo-infected” state, against which the immune system mounts a response. Because RRx-001 is immunomodulatory but lacks the toxicity of standard chemotherapeutics, epigenetic agents, and checkpoint inhibitors, it has the potential to amplify their activity without increasing toxicity.

## Abbreviations

5-AZA: Azacytidine
COBRA: Combined bisulfite restriction analysis
dsRNA: Double-stranded RNA
ERV: Endogenous retrovirus
FDA: Food and Drug Administration
GRCF: Genomic resources core facility
IFN: Interferon
ISGs: Interferon-stimulated genes
LTR: Long terminal repeat
qPCR: Quantitative polymerase chain reaction
STR: short tandem repeat
